# Food safety-oriented climate adaptation strategies in pastoral dairy systems of developing countries: A scoping review

**DOI:** 10.1016/j.onehlt.2026.101540

**Published:** 2026-08-16

**Authors:** Jacob Koome, Samuel Imathiu, Johnson Mwove, Monicah Maichomo, Mohammed Hussen Alemu, Dagim Belay, Søren Bøye Olsen, Jørgen Dejgård Jensen, John Kinyuru

**Affiliations:** a Department of Food Science and Technology, Jomo Kenyatta University of Agriculture and Technology, Nairobi, Kenya; b Department of Food Technology, Chuka University, Chuka, Kenya; c Department of Veterinary Sciences, Kenya Agricultural and Livestock Research Organization, Nairobi, Kenya; d Department of Food and Resource Economics, University of Copenhagen, Copenhagen, Denmark

**Keywords:** Chemical hazards, Climate adaptation, Climate change, Food safety, Microbial hazards, One Health, Pastoral dairy systems, Zoonoses

## Abstract

Pastoral dairy systems provide livelihoods for millions of inhabitants of arid and semi-arid lands (ASALs) in developing countries. However, these systems are threatened by adverse climate change. Complex and erratic rainfall patterns, increasing temperatures, and more frequent droughts are not only reducing milk production but also exacerbating risks to its safety. This scoping review synthesizes evidence from peer-reviewed scientific articles published between 2000 and 2025, retrieved from publicly available databases, to examine the effects of climate stressors on milk safety in smallholder pastoral dairy systems in the ASAL regions of Africa and Asia. The review finds that the most commonly reported climate-related risks are drought (37.5%), rainfall variability and heat stress (30%), and flooding (7.5%). These stressors contribute to extensive microbial contamination, including *Escherichia coli, Staphylococcus aureus, Salmonella spp*., and *Brucella spp*., as well as chemical hazards such as aflatoxins and antibiotic residues which constitute food safety challenges that should be addressed within a One Health framework. A combination of technological, ecological, behavioral and indigenous approaches has been employed as adaptation strategies to reduce climate change impacts. These include solar-powered milk cooling, rainwater harvesting and borehole development, herd diversification into drought-resistant species, and traditional smoke fumigation of milk containers. Additional innovations, such as rapid on-farm diagnostics, aflatoxin biocontrol, and training of milk handlers, also show potential for improving climate change adaptation. Nonetheless, adaptation interventions remain fragmented, hazard-specific, and insufficiently integrated into broader food safety frameworks. These findings imply that prioritizing milk safety within climate adaptation strategies could substantially safeguard public health and sustain pastoral livelihoods amid increasing climatic uncertainty.

## Highlights

• Climate change threatens pastoral dairy systems, reducing milk production and safety.
• Drought, heat stress, rainfall variability, and flooding increase milk contamination risks.
• Microbial and chemical hazards affect One Health.
• Adaptation strategies combine technology, ecology, behavior, and indigenous practices.
• Integrating milk safety with adaptation strategies safeguard public health.

## Graphical abstract

**Figure:**
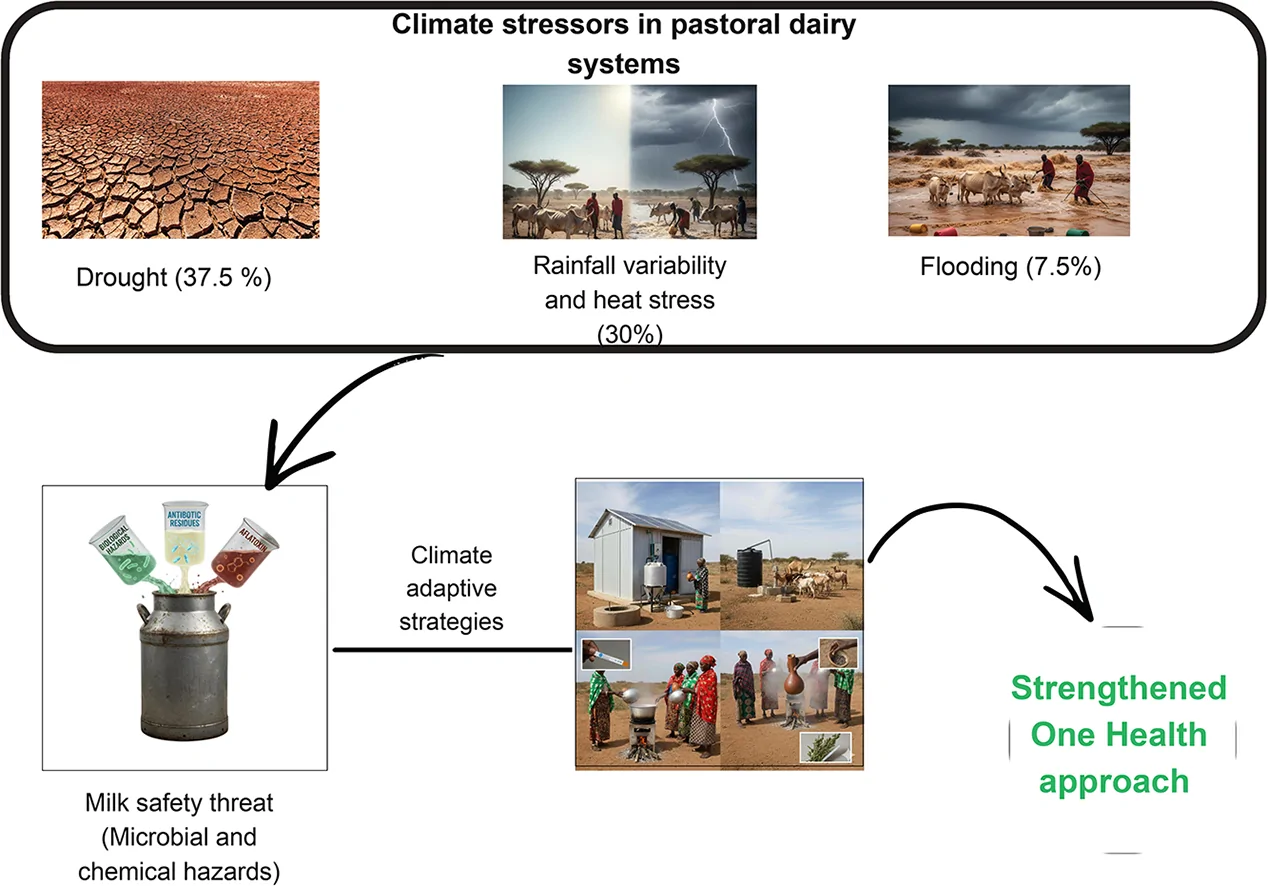

## 1 Introduction

Pastoralism is defined as a livelihood and production system in which at least 50% of household gross revenue is derived from livestock and related activities, and in which households rely on the management and movement of livestock across landscapes in response to seasonal variations in the availability of pasture and water [1], [2]. In a One Health approach, pastoral systems represent a complex human, animal and environment interconnected relationship, where livestock health, environmental factors and human health are inseparable and hence require the implementation of integrated approaches to disease prevention, food safety, and climate resilience [3], [4], [5], [6]. It represents a fundamental and often sustainable approach to land use, particularly prevalent in ASALs [1], [7], [8]. Globally, rangelands cover about 50–54% of the earth's terrestrial surface and are found in over 100 countries, supporting pastoralism and related land-based livelihoods [9], [10], [11], [12]. Approximately 200 to 500 million people practice pastoralism worldwide [13]. In Africa, pastoralism spans approximately 43% of the continent and supports about 268 million people [14]. It also makes a substantial contribution to agricultural and national economies, for example, accounting for up to 84% of agricultural gross domestic product (GDP) in Niger, 27% of national GDP in Chad, and 12% of national GDP in Mongolia [1], [13]. Beyond its economic role, pastoralism is integral to socio-cultural wellbeing, the preservation of ecosystem services, maintenance of rangeland biodiversity, and carbon sequestration [12], [13], [14]. In Kenya, communities primarily inhabiting the ASALs, such as the Maasai, Samburu, Turkana and Borana hold over 60% of the national livestock herd [15], [16]. These livestock systems significantly contribute to household food and nutrition security, especially through milk [17], [18], [19].

Pastoral systems are, however, increasingly imperiled by climate change, which amplifies existing vulnerabilities stemming from demographic, economic and political pressures [15], [20], [21], [22], [23]. Climate change is characterized by global alterations in weather patterns, notably rising average temperatures, erratic precipitation, and an increased frequency and intensity of extreme weather events such as droughts and floods [18], [19], [24], [25]. These climatic shifts directly undermine the complex mobility strategies and social networks that pastoralists have traditionally relied upon to cope with environmental variability, while also affecting food security [15], [26], [27], [28]. For instance, Kenya experienced its most severe drought in four decades between 2020 and 2023, marked by five consecutive failed rainy seasons. This drought disproportionately affected ASALs and forced pastoralists into longer and more distant migration routes [29].

These climatic stressors also have direct implications for food safety within pastoral dairy value chains [30]. Drought and water scarcity compromise hygiene practices, increasing the risk of microbial contamination [31], [32], [33]. Heat stress accelerates microbial growth during milk storage and long-distance transport, particularly in the absence of cooling infrastructure [34], [35]. Rainfall variability often results in unstable fodder availability, driving reliance on mold-contaminated feed and increasing the likelihood of exposure to mycotoxins such as aflatoxins, through milk consumption [36], [37], [38]. Flooding events may also facilitate the spread of zoonotic pathogens, including *Brucella* and Rift Valley fever, further threatening milk safety [15], [18]. Consequently, climate stressors not only affect milk safety but also exacerbate food safety risks in pastoral dairy systems [15], [39]. Hence, without paying close attention to food safety in these dairy systems under climate conditions within a One Health framework, it is difficult to solve these food safety challenges [4], [6], that is, to achieve an integrated, unifying approach to sustainably balance and optimize the health of people, animals, and ecosystems. Therefore, the aim of this scoping review is to map and synthesize the existing body of literature on food safety risks and climate adaptation strategies within pastoral dairy systems in Kenya and comparable ASAL regions of Africa and Asia. The review does not focus on developed countries due to differences in socioeconomic contexts, agro-climatic conditions, and the level of advancement in food safety control systems. It also excludes developing countries outside Africa and Asia because the majority of pastoral dairy systems are located within these regions. Asia hosts large pastoral and agro-pastoral dairy systems, particularly South and Central Asia, which face challenges similar to those in Africa, including climate variability, gaps in food safety knowledge and regulation and limited cold-chain infrastructure. This provides valuable comparative insights and transferable adaptation strategies that are relevant to African countries such as Kenya. Specifically, the review seeks to identify climate-related stressors that influence milk safety in pastoral dairy systems and document adaptation strategies that have been implemented or proposed to enhance food safety and sustainability in pastoral milk production. Accordingly, the review is guided by three key research questions: 1) What are the documented food safety risks in pastoral dairy systems in Kenya and comparable regions in Africa and Asia? 2) How do climate variability and environmental stressors compound these risks? 3) What adaptation strategies are currently being employed or recommended to mitigate these challenges?

## 2 Methodology

**Fig. 1:**
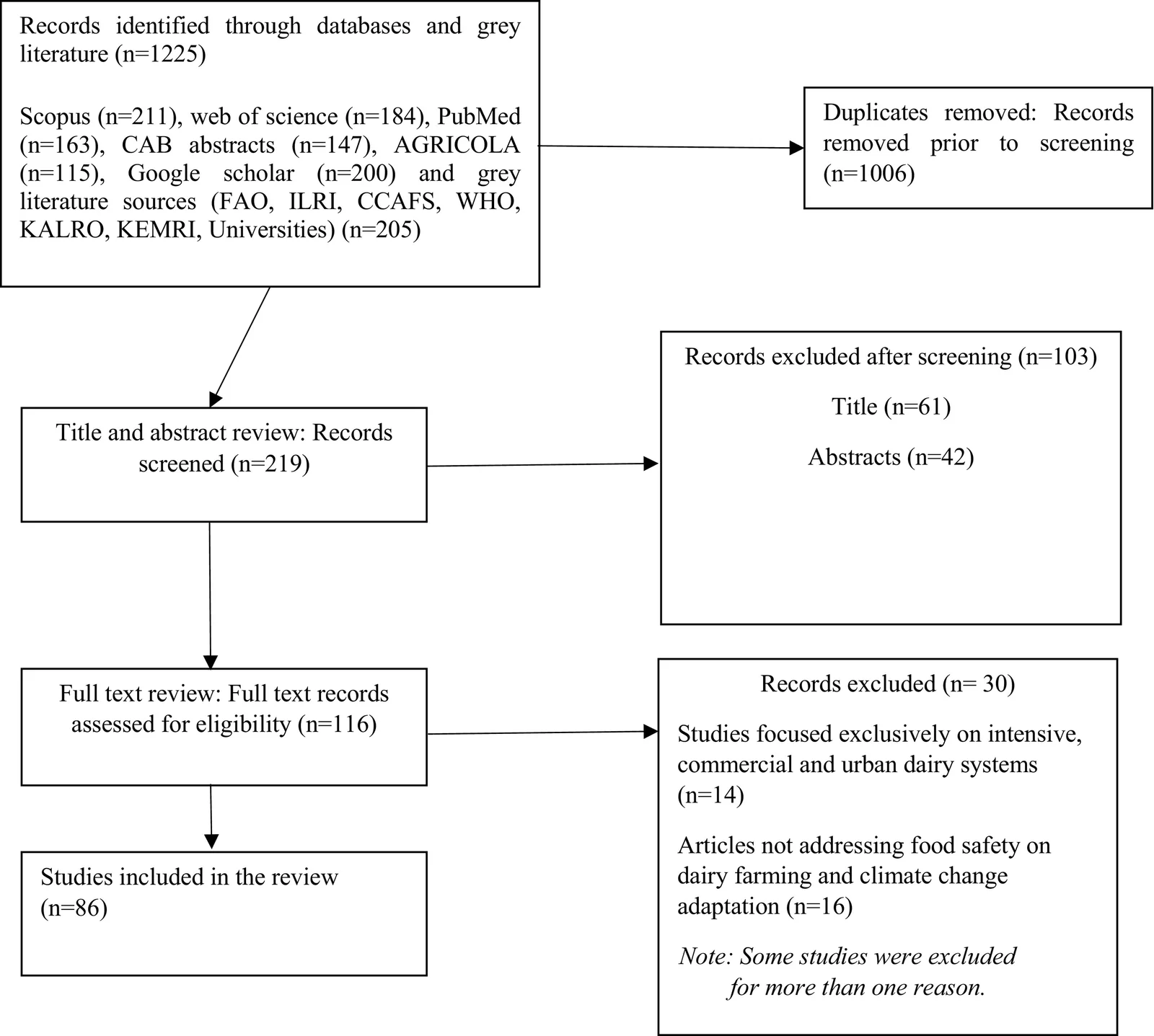
PRISMA-ScR flowchart of studies selection process.

A scoping review approach was used to systematically map and synthesize the diverse and fragmented evidence from the included studies. This approach was chosen because the available literature is highly heterogeneous and dispersed across multiple research domains, and because research on the nexus between climate change and food safety within pastoral dairy value chains is limited [40], [41]. The methodological and reporting standards outlined in the PRISMA extension for scoping reviews (PRISMA-ScR) were followed in conducting and reporting this review (Fig. 1) to ensure transparency and reproducibility [42].

### 2.1 Search strategy

A structured literature search was conducted between 20 June and 17 July 2025 across six major databases: Scopus, Web of Science, PubMed, CAB Abstracts, AGRICOLA, and Google Scholar. The search covered literature published from January 2000 up to July 2025 to capture a broad range of peer-reviewed and grey literature in the fields of agriculture, veterinary science, food systems, and environmental change. In addition to database searches, grey literature was sourced from institutional websites involved in agriculture and food systems research and policy, including the Food and Agriculture Organization (FAO), the International Livestock Research Institute (ILRI), the CGIAR Research Program on Climate Change, Agriculture and Food Security (CCAFS), the World Health Organization (WHO), the Kenya Agricultural and Livestock Research Organization (KALRO), the Kenya Medical Research Institute (KEMRI), and selected universities that host relevant research outputs. A systematic search strategy with keywords and thematic terms was employed, including, but not limited to, food safety, milk contamination, pastoral systems, climate variability, adaptation strategies, and dairy value chains. A single search string combining terms for climate stressors, milk safety hazards, and pastoral systems (e.g., (“climate change” OR “drought” OR “temperature” OR “rainfall variability”) AND (“milk safety” OR “milk contamination” OR “bacterial prevalence” OR “mycotoxins” OR “zoonoses”) AND (“pastoralism” OR “pastoralists” OR “smallholder dairy” OR “Africa” OR “Asia”)), adapted to the indexing rules of each database, was used to ensure consistency across searches. Reference lists of high-quality studies identified during the screening process were examined to identify additional relevant literature. Both backward citation and forward citation tracking were conducted to ensure the search was comprehensive.

### 2.2 Study eligibility and screening

A systematic eligibility assessment and screening procedure was employed to guide the study selection process. The review followed the Population, Concept, Context (PCC) framework, as recommended by the Joanna Briggs Institute Reviewers [43], to ensure that the inclusion criteria aligned with the scope of the review. The main aim was to identify studies that provide detailed analysis of food safety issues related to milk production in pastoral dairy systems that are simultaneously under pressure from climate change. The eligibility criteria are summarized in Table 1.

**Table 1:** Population, Concept, Context (PCC) framework for eligibility criteria for scoping review.

Domain	Inclusion criteria	Exclusion criteria
Population (P)	Pastoral and nomadic communities located in Kenya, Africa, and Asia	Studies dealing only with intensive, commercial, or urban dairies
Concept (C)	Food safety issues, such as milk contamination, microbial quality, dairy hygiene, and climate adaptation strategies	Articles not related to food safety or climate adaptation
Context (C)	Arid and semi-arid regional areas that include pastoral dairy systems	Studies from regions outside Africa or Asia unless directly relevant
Type of Source	Peer-reviewed articles, academic theses, NGO and government reports with empirical or policy aspects	Editorials, blog articles, and commentaries with no evidence-based information
Time frame	Publications from 2000 to 2025	Literature published before 2000, except when cited in current policy literature
Language	English	Non-English language publications

Ref.: [43].

The search yielded a total of 1225 records from academic databases and grey literature sources. After importing these records into the Mendeley reference manager, 1006 duplicates were removed, leaving 219 records before screening. Titles and abstracts were screened for relevance based on the PCC criteria, resulting in exclusion of 103 studies. The remaining 116 were retrieved in full and assessed for eligibility. During this stage, 30 studies were excluded; 14 focused on intensive or commercial dairy systems, and 16 did not address food safety or climate adaptation. Consequently, 86 studies were retained and included in the final review. The full texts of all included articles were accessed and analyzed. Taken together, these studies form the empirical foundation of the review, providing up-to-date evidence on food safety hazards, milk hygiene measures, and climate adaptation in pastoral dairy systems across Africa and Asia. The screening process is documented using a PRISMA-ScR flow diagram in Fig. 1.

### 2.3 Data extraction

Data extraction was conducted using a predetermined and systematically applied extraction framework. Data was charted across the following variables: year of publication, country, dairy system type, climate stressors, milk safety risks, climate adaptation strategies, study design, source type, and key findings. This approach enabled the consistent capture of both quantitative and qualitative information from the included studies. All data were recorded using structured templates to ensure uniformity, categorical variables were coded, and narrative information was extracted and documented in detail.

## 3 Results

### 3.1 Number of included studies and year of publication

**Fig. 2:**
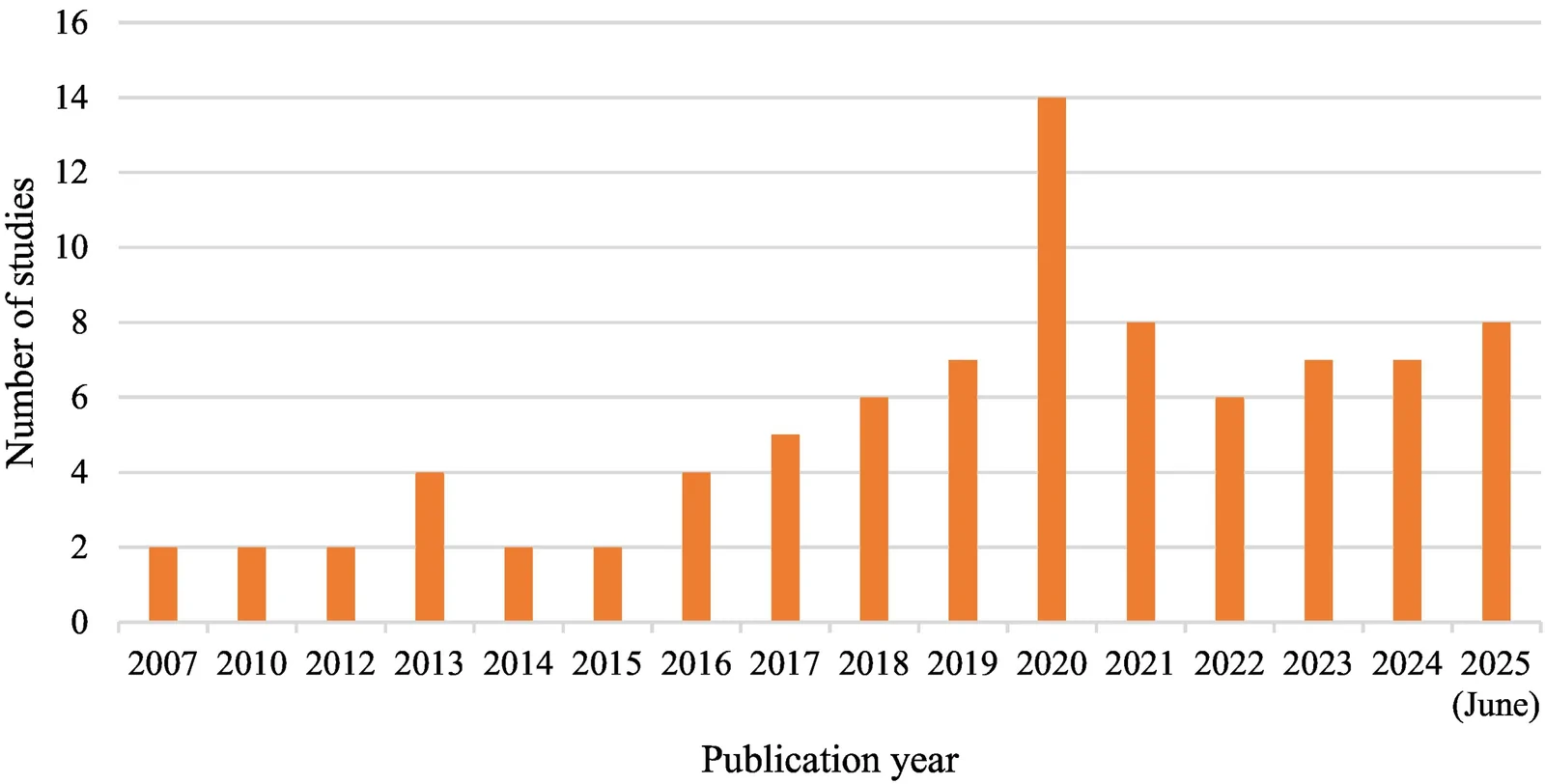
Year of publication of the included studies (*n* = 86).

The studies included in this review (*n* = 86) were published between 2007 and July 2025. The trend of their publication is illustrated in Fig. 2 showing a peak in the year 2020. Kenya accounted for the highest proportion of studies (37.2%), followed by Ethiopia (18.6%). The fewest studies were conducted in Pakistan, Senegal, Zambia, Tanzania, Mali, South Sudan, Malaysia and Mongolia.

### 3.2 Typology of pastoral dairy systems in Africa and Asia

Out of 86 studies included, 48.8% (*n* = 42) focused on pastoral cattle systems. Camel pastoral systems were examined in 20.9% (*n* = 18) of the studies, while mixed smallholder systems, combining cattle, goats, and camels were examined in 30.2% (*n* = 26).

### 3.3 Dominant study designs and types of literature sources

The methodological composition of the studies included in this scoping review indicates that empirical research approaches are dominant. Quantitative methods constituted the largest share with 36 studies. Mixed-method designs were also well represented, comprising 27 studies. Review articles accounted for 23 studies, of which 21 were peer-reviewed and two were classified as grey literature.

### 3.4 Comparative prevalence of milk contamination in Africa and Asia

**Fig. 3:**
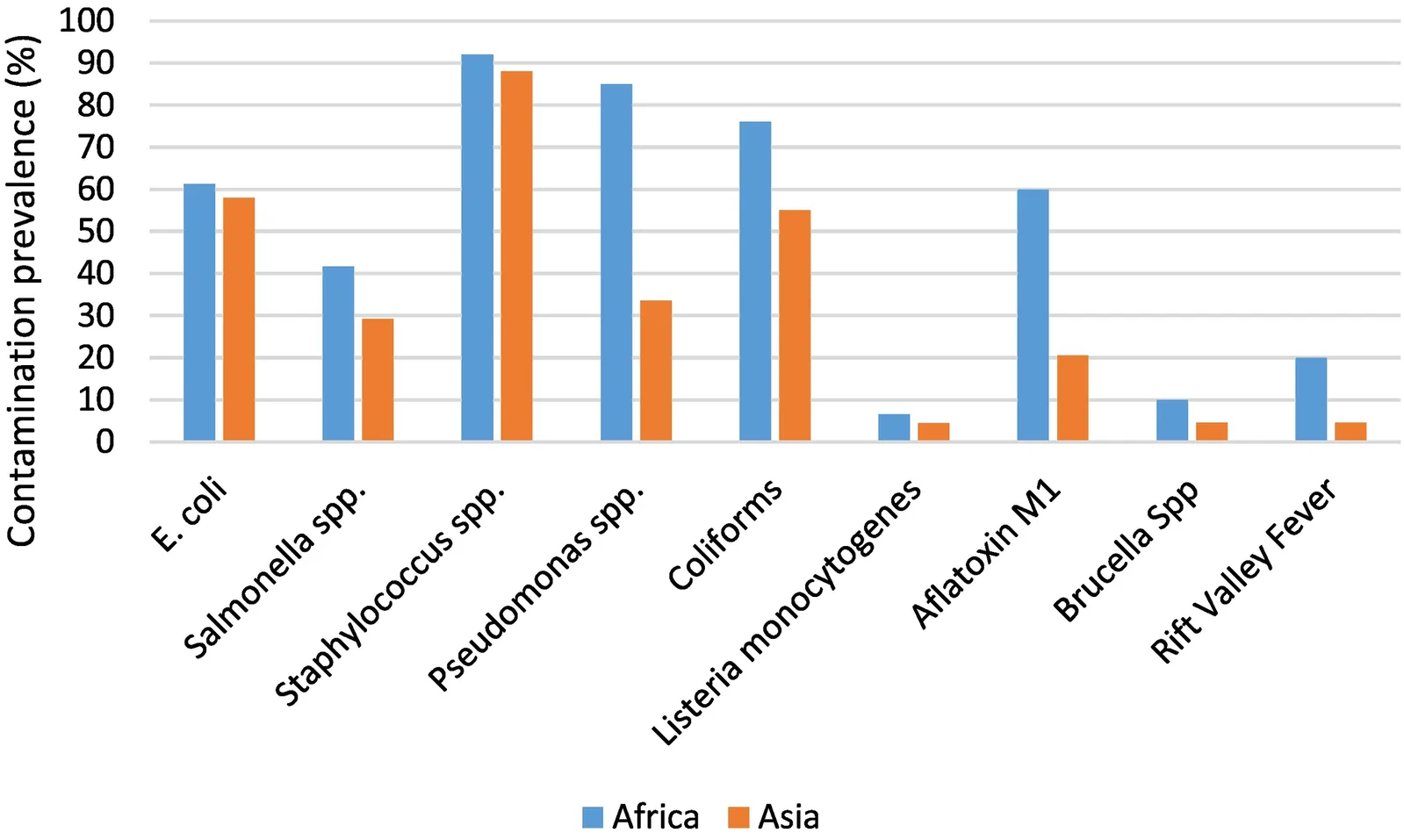
Comparative prevalence of major hazards that contaminate milk in African and Asian pastoral dairy systems. (*n* = 13).

Although direct comparison of milk contamination prevalence between Africa and Asia is constrained by socio-economic, infrastructural, climatic, and political differences between the two regions [1], [2], [7], [18], we present comparative results to provide an indicative overview of milk contamination prevalence in developing countries. This broadens the evidence base. Milk safety concerns appear to be more pronounced in Africa than in Asia's pastoral dairy systems, as indicated by differences in contamination levels shown in Fig. 3.

In Africa, *E. coli* contaminated 61.2% of the samples analyzed, compared with 58% in Asia [55], [58], [60], [63], [65], [75], [76]. *Salmonella* spp. was present in 41.7% of the African samples and approximately 29.2% of Asian samples [56], [58], [59], [63], [65]. *Staphylococcus* spp. was the most prevalent hazard, detected in 92% of samples in Africa and 88% in Asia [34], [35], [56], [58], [60], [63], [65]. *Pseudomonas* spp. was detected in 85% of African samples, compared with 33.5% in Asia [35], [63]. Coliforms were observed in 76% of African samples and 55% of Asian samples [32], [55], [57], [60], [67]. *Listeria monocytogenes* was found in 6.5% of samples in Africa and 4.35% in Asia [34], [35], [58], [59]. Regarding chemical hazards, Aflatoxin M1 was detected in approximately 60% of milk samples in Africa, compared with 20.5% in Asia [74], [92], [93]. Zoonotic pathogens such as *Brucella* spp. had an average seroprevalence of approximately 10% in Africa and 4.5% in Asia [69], [70]. Moreover, Rift Valley fever was reported at a seroprevalence of approximately 20% in Africa, whereas in Asia only sporadic cases were recorded, with an estimated prevalence of about 4.5% [18], [24], [72].

### 3.5 Climate stressors and associated food safety risks

Climate stressors were explicitly mentioned in 40 of the 86 included studies. Out of 40 studies, as indicated in Table 2, drought, together with water scarcity, and rainfall variability combined with high temperatures were the most frequently reported climate stressors. Studies addressing drought predominantly identified milk spoilage-related losses and accelerated bacterial proliferation, with frequent detection of *E. coli* and *Salmonella* spp. [18], [24], [32], [58], [62]. Rainfall variability and high temperature were associated with elevated coliform counts, high somatic cell counts (SCC), and increased prevalence of *E. coli*, *Pseudomonas* spp., and *Staphylococcus* spp., with over 90% of milk samples reported as microbiologically unsatisfactory [24], [28], [35], [60], [63]. Flood events were linked to an increased risk of zoonotic pathogen transmission [5], [19], [20]. Notably, several studies did not explicitly link food safety risks to specific climate stressors. Within this group, the presence of Shiga-toxin-producing *E. coli* (STEC), aflatoxins, and antibiotic residues was reported; however, these findings were typically discussed without a direct connection to underlying climatic drivers [73], [74], [77].

**Table 2:** Distribution of studies by climate stressor and top ranked food safety risk.

Climate stressor	Number of studies (*n* = 40)	*n* (%)	Identified risks	References
Drought and water scarcity	15	37.5%	Milk spoilage losses and accelerated bacterial proliferation; *E. coli*, *Salmonella* spp.	[18], [21], [22], [24], [26], [31], [32], [38], [39], [57], [58], [61], [62], [64], [68]
Rainfall variability and high temperature	12	30.0%	Elevated coliform counts and high SCC; *E. coli*, *Pseudomonas* spp., *Staphylococcus* spp., TVC >5.81 log CFU/mL	[27], [28], [33], [34], [35], [55], [56], [60], [63], [65], [66], [67]
Flood events	3	7.5%	Increased transmission of zoonotic pathogens	[5], [19], [20]
Not well mapped	10	25.0%	Shiga-toxin producing *E. coli,* aflatoxin occurrence, antibiotic residues	[34], [37], [73], [74], [75], [76], [77], [89], [92], [93]

Note: SCC = Somatic Cell Count, TVC = Total Viable Count, CFU = Colony Forming Units.

### 3.6 Climate-adaptation measures to mitigate food safety risks

Drought and water scarcity were associated with bacterial proliferation and milk spoilage-related losses, particularly in regions lacking adequate cold-chain facilities [86], [88]. A range of adaptation measures has been reported in these areas. These include improved hygiene of milk containers, provision of shade to protect milk during daytime handling, and traditional food preservation methods such as smoke fumigation of milk storage calabash gourds [31], [79], [80], [83], [89]. Technological and infrastructural interventions have also been documented, including solar-powered water pumps, rainwater harvesting systems, and borehole development to improve water access [16], [29], [39], [53]. Diversification of herd composition was reported as an additional climate adaptation strategy, involving the use of heat- and drought-tolerant species such as camels, goats, and Sahiwal cattle [20], [48], [49], [54]. Furthermore, the use of solar-powered coolers and vehicles equipped with cold-chain facilities along the dairy value chain was recommended to curb bacterial proliferation and reduce high rates of milk spoilage [81], [86], [87]. Livestock vaccination campaigns and programs were also identified as an effective adaptation approach in areas that are highly affected by floods, particularly for mitigating zoonotic diseases and reducing transmission risk [18], [19], [46]. In addition, hazards such as aflatoxin, STEC, and antibiotic residues were reported to be mitigated through targeted interventions. These included on-farm Loop-mediated isothermal amplification (LAMP) diagnostic kits for early detection of milk-borne and zoonotic pathogens, batch-level micro-pasteurization, improved forage drying and storage using hermetic bags, and the application of biocontrol agents such as atoxigenic *Aspergillus* strains to suppress aflatoxin production in dairy feed [34], [74], [78], [92], [93]. To control and prevent antibiotic residues in milk, farmer training on appropriate withdrawal periods following cattle treatment was recommended [34], [37], [89]. However, some measures were commonly reported as being used across many pastoral areas, as shown in Table 3.

**Table 3:** Frequently reported climate adaptive strategies in pastoral dairy systems.

Adaptation strategy	Number of studies (*n* = 31)	*n* (%)	Representative implementation setting	References
Food grade containers with container hygiene and shading	8	25.8%	Isiolo (Kenya) camel value chains	[31], [32], [55], [79], [80], [83], [89], [90]
Training and narrative risk communication	4	12.9%	Maasai storytelling sessions; workshops in Senegal	[34], [37], [84], [89]
Traditional smoking of milk containers	3	9.7%	West Guji (Ethiopia) pastoral campsites	[31], [80], [83]
Solar powered coolers at aggregation centres and on transport vehicles	5	16.1%	Western Zambia; Kenyan smallholder milk cooperatives	[67], [81], [86], [87], [90]
Water management; solar pumps, rainwater harvesting and boreholes	6	19.4%	Solar-driven boreholes for dairy goats in Marsabit County (Kenya); rooftop catchment in Kajiado (Kenya)	[16], [29], [39], [53], [71], [94]
Diversification of herd and genetic improvement through camels, goats and cattle	5	16.1%	Introduction of camel milking among the Samburu (Kenya); Sahiwal–indigenous crossbred trials in Machakos (Kenya)	[15], [20], [48], [49], [54]

## 4 Discussion

### 4.1 Overview of pastoral dairy systems in Africa and Asia

This scoping review demonstrates clear temporal shifts in research on pastoral areas across Africa and Asia. Publication volume has accelerated significantly, with 83.7% of the included studies published since 2016, reflecting growing recognition of climate change's impacts on pastoral dairy food safety and adaptation. While this represents a positive development, research remains unevenly distributed across regions, with most studies concentrated in a few countries, like Kenya and Ethiopia, and limited evidence available from many pastoral areas. The volume of research has increased over the past two decades, likely due to growing awareness of climate change as a threat to food safety and livelihoods in ASALs. More broadly, global interest in resilience building, manifested through climate action initiatives such as the Paris Agreement and regional adaptation programs in East Africa, has likely contributed substantially to this growth [44], [45], [46]. However, these patterns may also reflect the concentration of research funding and institutional capacity in certain regions, rather than alignment with areas where research needs are greatest to effectively address impact of climate change. The large number of studies in Kenya might have been influenced by the country's relatively developed dairy sector, strong research infrastructure, and the presence of national and international research organizations including KALRO and ILRI, that are actively involved in livestock research. Nonetheless, this dominance creates a geographic imbalance, with disproportionate emphasis on Kenyan pastoral areas. This carries the risk of portraying Kenyan systems as representative of the entire African region, despite substantial differences in social, ecological and market conditions.

The growing prominence of Ethiopia is attributed to increasing engagement with pastoral areas such as Borana, Somali and Afar, where research on food safety and climatic adaptation is also expanding. In contrast, countries such as Pakistan, Senegal, Zambia, Tanzania, Mali, South Sudan and Mongolia remain underrepresented, despite facing similar, in some cases more severe, challenges related to drought, heat stress, and water scarcity. Limited research coverage in these regions may stem from logistical challenges associated with conducting fieldwork among highly mobile and hard-to-reach pastoral communities, where insecurity and inadequate road infrastructure can hinder systematic data collection. Therefore, while we present comparative evidence on milk contamination prevalence between Africa and Asia, we acknowledge that such comparisons should be interpreted with caution given these contextual differences.

Three main types of pastoral dairy systems are reported in the literature: cattle-based, camel-based and mixed livestock systems. The majority of the studies focus on pastoral dairy systems that involve cattle pastoralism. In East Africa, cattle pastoralism has historically and economically played a significant role in pastoral livelihoods [10], [37], [47]. Camel systems were, however, examined in only one fifth of the reviewed studies, despite their increasing importance in many ASAL regions of Africa and Asia [48], [49]. Mixed systems, which combine cattle, goats and camels, appear to receive attention comparable to cattle-based systems. This pattern indicates that research has been heavily skewed toward cattle-based dairy systems, reflecting their dominance in national dairy economies and the focus of dairy development programs in many regions [38], [50], [51]. The limited investigation of camel and mixed systems overlooks their adaptability advantages under shifting climatic conditions, as camels are highly resistant to heat, drought, and feed scarcity [20], [26], [52].

Camels and small ruminants are increasingly being incorporated as important household milk sources in the Horn of Africa and parts of Pakistan due to rangeland degradation and increasingly unpredictable rainfall pattern [1], [21]. Overall, the findings indicate a paucity of published knowledge on camel and mixed dairy systems. While cattle remain the cornerstone of pastoral dairy research, the evolving realities of herd diversification and ecological adaptation are not yet fully reflected in the scientific literature [20], [53]. Notably, these topics are more extensively covered in grey, unpublished literature. A more balanced research agenda that includes camel and mixed systems would enhance pastoralists' capacity to plan production strategies that sustain livelihoods and milk safety under climatic change [46], [54]. Such evidence would also support more informed investment decisions by governments and development partners.

Methodologically, the reviewed studies rely primarily on quantitative observational and mixed-methods research designs, reflecting a growing body of evidence on climate–food safety interactions in pastoral dairy systems. However, the relative paucity of longitudinal studies highlights the need for long-term, context-specific studies that would trace the progress of adaptation practices in response to environmental and socioeconomic changes. While quantitative studies and literature reviews provide valuable evidence, they also highlight a methodological gap due to the limited number of primary qualitative and mixed-methods studies. Further studies would benefit from stronger triangulation of empirical evidence through participatory and co-designed approaches that better capture the lived experiences of pastoral smallholder dairy farmers, particularly women and marginalized herders, who are underrepresented in the pastoralism literature. Such approaches could contribute to reducing milk contamination in both regions, with potentially greater impact in Africa.

The reviewed studies suggest that milk contamination may be more prevalent in African pastoral dairy systems than those in Asia. In both regions microbial contamination was reported more frequently than chemical contamination. This pattern may indicate greater awareness of, and adherence to food safety practices along the value chain among Asian pastoralists compared with their African counterparts. Asian pastoral dairy farmers may also benefit from stronger government or non-governmental support, including access to cold-chain facilities, training in milk hygiene and handling practices, improved sanitation, and more reliable sources of clean water. These factors may contribute to lower level of milk contamination along the value chain. However, it is important to note that a larger proportion of the reviewed studies were conducted in Africa, which may introduce bias in observed regional differences in contamination levels. In practice, lower observed contamination rates in certain Asian pastoral contexts could be reflecting the greater integration of decentralized milk collection centers, broader availability of basic solar cooling units, and established dairy cooperative extension models. Conversely, higher microbial contamination reported in African pastoral value chains underscores an acute operational deficit in clean water for equipment washing at mobile campsites.

### 4.2 Climate impact on the food safety in pastoral dairy systems

The climate-related stressors identified in the reviewed studies were interconnected and contributed to a range of food safety risks. Drought was consistently reported to increase microbial contamination, leading to milk spoilage and poor hygiene due to water scarcity [31], [32], [34], [35], [55]. This is because water is essential for maintaining good hygiene practices throughout milk production and across the entire value chain. Microbiological analyses frequently reported the presence of *E. coli* which is associated with poor hygiene and *Salmonella* spp., indicating that the limited water access constrains the cleaning of milking and storage containers, while elevated temperatures accelerate bacterial proliferation during milk storage and long-distance transport [56], [57], [58], [59]. These findings illustrate how chronic climatic stress exacerbates poor hygiene conditions, thereby amplifying contamination risks in ASAL regions where cold-chain facilities are either unavailable or difficult to maintain [60], [61], [62]. Similarly, rainfall variability and elevated temperatures were associated with increased coliform and somatic cell counts [63], [64], [65], alongside widespread detection of *E. coli*, *Pseudomonas* spp., and *Staphylococcus* spp. [31], [55], [63], [66], [67]. These findings suggest that heat stress and rainfall fluctuations not only compromise livestock health but also degrade post-harvest milk quality [68]. Overall, the evidence indicates that pastoral households face compounded food safety risks when climate-induced livestock stress coincides with poor hygiene conditions.

Although limited in number, studies addressing flood revealed alternative pathways through which climate change undermines milk safety in the pastoral regions. Heavy rains and floods were associated with the spread of zoonotic pathogens via contaminated surface runoff and shared groundwater sources used by both livestock and pastoralists [[5], [19], [20]]. Despite their importance for One Health risk management, such acute climatic events remain underrepresented in the literature, which has tended to focus heavily on chronic stressors such as drought [71]. Moreover, only a few studies reported hazards such as Shiga toxin-producing *E. coli*, aflatoxins, and antimicrobial residues, and these studies did not clearly link their occurrence to specific climate stressors [24], [34], [36], [72], [73], [74], [75], [76].

This highlights a recurring limitation in pastoral dairy research, the tendency to treat food safety and climate change as parallel rather than integrated challenges [16]. Consequently, without explicitly linking hazard occurrence to climatic variability, such as temperature changes, altered rainfall patterns, or vector dynamics, the field remains limited in its capacity to model causal pathways and develop effective, sustainable adaptation strategies.

### 4.3 Integrated climate adaptation strategies to mitigate food safety risks

Mitigating the food safety risks in pastoral dairy systems in the context of climate change requires integrated and holistic approaches. As reported in the reviewed studies, these measures span across traditional practices and technology-driven innovations [21], [77], [78], [79]. Behavioral strategies, such as improved container hygiene, shading milk during collection and transport, and smoke fumigation of milk gourds, were widely reported as effective means of ensuring milk safety and reducing losses along the dairy value chain both in general and climate-affected contexts [80], [81], [82], [83]. These approaches rely on low cost, culturally embedded practices to maintain milk safety in resource-constrained pastoral settings and are therefore widely adopted [47], [84]. However, their effectiveness varies considerably and remains poorly documented, underscoring the need for systematic evaluations of the safety and efficacy of traditional milk preservation methods.

Technological approaches focus on enhancing water availability, predicting pasture availability and improving cooling facilities [46], [85]. Studies reported the adoption of solar-powered water pumps, rainwater harvesting systems, and borehole construction as important interventions for enabling hygienic milking and effective container sanitation along the pastoral dairy value chain [15], [20], [25]. Similarly, the use of solar powered coolers and milk transport vehicles equipped with cooling facilities was cited as a means of controlling microbial growth by maintaining low milk temperatures throughout the value chain [56], [86]. However, high capital investment, installation costs, and ongoing maintenance requirements, challenge the sustainability and scalability of these technologies in pastoral dairy systems [86].

Livestock diversification and breeding improvements were also suggested as adaptive measures that may indirectly enhance milk safety through biological pathways [15], [47], [52], [87]. The integration of heat- and drought-tolerant breeds, such as camels, Sahiwal cattle and goats, was reported as an adaptation strategy to reduce disease incidence under climate change [48], [49]. Reduced disease burden may, in turn, decrease reliance on antibiotics, thereby lowering the risk of antimicrobial residues in milk and mitigating the spread of antimicrobial resistance among consumers [46], [48], [49], [88]. Training on antibiotic withdrawal periods following livestock treatment was also identified as an important behavioral intervention for reducing antibiotic residues in milk [81], [89], [90], [91]. In the context of climate change, rising temperatures and shifting rainfall patterns increase livestock disease burdens and heat stress, driving higher antibiotic use by pastoralists, therefore, targeted training ensures that increased usage does not compromise food safety through non-compliant withdrawal times. However, most of these interventions remain at the pilot stage and are confined to specific geographical contexts, highlighting the need for region-specific breeding and adaptation programs that respect local traditional knowledge systems and cultural practices [52].

Chemical hazards and zoonotic risks received limited attention in the reviewed studies, although several promising mitigation efforts were reported. These included the use of on-farm rapid diagnostic tools, such as LAMP assays for STEC detection, batch-level micro-pasteurization for pathogen elimination, and biological control methods such as atoxigenic Aspergillus strains to mitigate aflatoxin contamination in dairy feeds [92], [93].

However, the implementation of these technologies remains uneven and challenging in ASALs due to broader infrastructural and development constraints. Overall, the reviewed studies indicate that adaptation strategies in pastoral dairy systems are fragmented and mostly designed to address specific hazards**,** often implemented in isolation rather than as components of integrated food safety and climate adaptation frameworks [16], [53], [94]. While technological and infrastructural innovations in Africa and Asia are gradually gaining traction, the continued reliance on traditional food safety practices, many of which have demonstrated resilience under climatic stress, suggests that effective adaptation will likely require blending indigenous knowledge with modern interventions [80], [81]**.**

## 5 Strengths and limitations of the study

This study represents the first scoping review to focus on food safety hazards in pastoral dairy systems of Africa and Asia in the context of climate change. In addition, it identifies available and applicable climate-adaptive strategies that support sustainable milk production in these systems. The review draws on heterogeneous sources, which is a key strength, as it enables the identification of recurring themes across African and Asian pastoral regions. The inclusion of review articles further enriched the synthesis; however, this may have introduced a degree of secondary-level bias. The primary focus of the review was to examine the impact of climate change on the food safety of pastoral dairy systems in Kenya, while drawing comparative insights from other pastoral dairy systems in Africa and Asia. Consequently, a higher concentration of studies originated from Kenya and the neighboring country of Ethiopia, which may limit the generalizability of the findings to the pastoral dairy systems across the entire African and Asian regions. In addition, only a limited number of studies explicitly examined correlations between food safety hazards and climate stressors. As a result, some thematic inferences were drawn from associations that were not always clearly or directly established. Moreover, the inclusion of studies employing diverse methodological approaches enhanced contextual understanding of pastoral systems but constrained both comparability and limited opportunities for quantitative synthesis. To address these limitations, future research should consider the following:

a) Design studies that explicitly link climate stressors, such as elevated temperature, droughts and flooding, to specific food safety hazards, including aflatoxin contamination and antibiotic residues to reduce conceptual and analytical fragmentation.
b) Complement scoping reviews with structured appraisal tools to assess the strength, quality and reliability of the available evidence.
c) Encourage targeted research in underrepresented pastoral regions to improve the balance and geographic coverage of the existing evidence base.
d) Adopt participatory and co-designed approaches that better capture the lived experiences of pastoral smallholder dairy farmers, particularly women and marginalized herders, who are underrepresented in the pastoralism literature

## 6 Knowledge gaps

It is evident from the growing body of climate change research that studies examining the impact of climate change on pastoral dairy systems are increasing. However, substantial knowledge gaps remain in both Africa and Asia. These gaps include: 1) Existing research is dominated by cattle-based pastoral systems, while camel- and goat-based systems remain underrepresented. This persists despite the greater resilience of camel and small ruminants to high temperature, water scarcity, and the harsher conditions characteristic of ASALs under climate change. Consequently, knowledge of milk-borne hazard profiles, consumer acceptance and the economic viability of milk from these species in the context of climate change remains limited, 2) There is limited mapping of groundwater availability across pastoral regions in Africa and Asia. This constrains the development of infrastructures for sustainable water supply and hampers the design of hygiene interventions that target specific food safety hazards at critical points along the pastoral dairy value chain, 3) Zoonotic diseases, such as brucellosis and Rift Valley fever, remain major concerns in pastoral dairy systems under variable climatic conditions. Longitudinal surveillance is therefore needed to generate evidence for the development of targeted and effective vaccination programs, as well as improved vector control strategies, which are essential for ensuring sustainable milk safety in pastoral dairy systems, and 4) New bacterial strains of potential food safety and public health concern may have emerged in the pastoral dairy systems due to the effects of climate change. Metagenomics analysis can be integrated into microbiological analysis of longitudinal studies to keep track of microbiological hazard profiles and guide necessary interventions.

## 7 Conclusion

African and Asian pastoral dairy systems are vital to the livelihoods of their inhabitants and food security, yet they remain highly vulnerable to food safety hazards that are increasingly exacerbated by climate change. As highlighted in this review, major sources of milk contamination include unhygienic milking practices, inadequate cooling systems, poor water quality and limited availability along the dairy value chain. These risks are further compounded by infrastructural limitations. Climate change intensifies these challenges by increasing water scarcity, deteriorating forage, and placing additional physiological stress on livestock, thereby elevating the risk of milk contamination. Without sustainable, context-specific adaptive interventions, these dynamics may undermine both public health and the resilience of pastoral communities. Integrated approaches offer dual avenues for adaptation and mitigation. To be effective, interventions must be supported by institutional commitment to strengthen rural veterinary and extension services, ensure equitable access to cold-chain facilities and clean water, and incorporate participatory, culturally appropriate co-design of food safety innovations. By integrating food safety interventions with sustainable climate adaptation strategies within a One Health framework, pastoral dairy systems can strengthen the interconnected health of humans, animals, and the environment. Such an integrated approach promotes healthier livestock, safer milk production, improved ecosystem resilience, and reduced risks of foodborne and zoonotic diseases, while protecting consumer health and safeguarding the cultural heritage and livelihoods of pastoral communities across Africa and Asia.

## CRediT authorship contribution statement

**Jacob Koome:** Methodology, Formal analysis, Conceptualization, Writing – original draft. **Samuel Imathiu:** Supervision, Methodology, Data curation, Conceptualization, Writing – review & editing, Resources, Funding acquisition. **Johnson Mwove:** Supervision, Methodology, Data curation, Conceptualization, Writing – review & editing. **Monicah Maichomo:** Methodology, Conceptualization, Writing – review & editing, Resources, Funding acquisition. **Mohammed Hussen Alemu:** Validation, Resources, Funding acquisition, Writing – review & editing. **Dagim Belay:** Validation, Writing – review & editing, Resources, Funding acquisition. **Søren Bøye Olsen:** Visualization, Validation, Writing – review & editing, Resources, Funding acquisition. **Jørgen Dejgård Jensen:** Validation, Supervision, Resources, Funding acquisition, Project administration. **John Kinyuru:** Resources, Methodology, Investigation, Conceptualization, Writing – review & editing, Funding acquisition.

## Funding

10.13039/501100011054Danish International Development Agency (DANIDA), 10.13039/501100005369Ministry of Foreign Affairs of Denmark, funded this research work through the ADAPTiVE project (Grant number: 24-M08-KU).

## Declaration of competing interest

This study was conducted without any financial ties or commercial purposes that could be interpreted as a possible conflict of interest. The authors declare that they have no conflicts of interest.

## Data Availability

The findings informing the conclusions of this scoping review were derived entirely from publicly available articles, reports and grey literature indexed in scientific databases. No primary data were generated by the authors. All extracted information is included within the manuscript and its references, and additional supporting materials are available from the corresponding author upon reasonable request.
